# Cognitive Testing of Items Measuring Movement Behaviours in Young Children Aged Zero to Five Years: Development of the Movement Behaviour Questionnaires for -Baby (MBQ-B) and -Child (MBQ-C)

**DOI:** 10.3390/children10091554

**Published:** 2023-09-14

**Authors:** Rebecca Byrne, Caroline O. Terranova, Li Kheng Chai, Denise S. K. Brookes, Stewart G. Trost

**Affiliations:** 1 School of Exercise and Nutrition Sciences, Faculty of Health, Queensland University of Technology, Centre for Children’s Health Research, Graham St., Brisbane 4001, Australia; ra.byrne@qut.edu.au (R.B.); caroline.terranova@qut.edu.au (C.O.T.); likheng.chai@qut.edu.au (L.K.C.); denise.brookes@qut.edu.au (D.S.K.B.); 2 School of Human Movement and Nutrition Sciences, The University of Queensland, Brisbane 4072, Australia

**Keywords:** infants, children, active play, physical activity, screen time, sleep, cognitive interviews

## Abstract

This paper describes the cognitive interview phase of the development of two brief surveys, the Movement Behaviour Questionnaire-Baby (MBQ-B) and Movement Behaviour Questionnaire-Child (MBQ-C), which measure the duration of physical activity, screen time, and sleep of children aged 0–5 years. The aims were (1) review the format, content, and clarity of questionnaire items and response options, (2) understand how parents retrieve, encode, and formulate responses when asked about their child’s movement behaviours, and (3) identify potential sources of response error and make appropriate modifications. Interviews with parents of children aged 0–5 years were conducted using concurrent think-aloud techniques and probing questions. Parents reviewed the MBQ-B and/or MBQ-C depending on the developmental stage of their child(ren). Twenty-nine interviews were conducted with 20 parents, over four iterative rounds. Participants recalled usual family routines and rules when estimating the duration/frequency of behaviours. To estimate active play, parents referred to the child’s daily routine considering wake and bedtimes, naps, and mealtimes. Participants were influenced by the examples provided, being unable to interpret these as exemplars only. Decomposing general items into specific questions with examples was well received. Use of numeracy skills when estimating duration was evident. Interviews informed revisions to item wording, examples, and recall prompts, which will be taken forward into the MBQ-B and MBQ-C validation studies. Utilising cognitive interviewing can enhance confidence that questionnaire items are correctly interpreted and understood by participants.

## 1. Introduction

Regular physical activity, limited screen time, and adequate sleep are movement behaviours inextricably linked to healthy growth and development during early childhood [1]. Achieving the optimal combination of physical activity, sedentary behaviour, and sleep is associated with a multitude of positive health outcomes. These include improved cardiometabolic and musculoskeletal health, and cognitive development, while being inversely associated with negative health outcomes such as obesity, elevated blood lipids, hypertension, and glucose intolerance [2,3].

On the weight of this evidence, numerous countries, including Australia, have developed 24-h movement guidelines for young children from birth to 5 years [4,5,6]. Infants can be physically active through supervised, interactive floor-based play including at least 30 min of tummy time, while toddlers and preschoolers are recommended to spend at least 180 min per day in a variety of physical activities [7]. For preschoolers, at least 60 min of this activity should be energetic play. Children of all ages should not be restrained for more than one hour at a time (e.g., in a stroller or car seat) and for those younger than two years, sedentary screen time is not recommended. While recommendations for sleep duration vary slightly with age, all have an emphasis on good quality sleep [5,6,7]. Such guidelines acknowledge that individual movement behaviours, including activity, sedentary time, screen time, and sleep, need to be considered in relation to each other when examining their associations with the health and developmental outcomes in children [4].

The ability to monitor population trends in meeting the 24-h movement guidelines and evaluate the impact of policies and programs to promote healthful lifestyle behaviours in children aged 0–5 years depends on the availability of validated short-form assessment tools. However, brief, validated, and ‘fit-for-purpose’ assessment tools for infants, toddlers, and preschoolers that are feasible for use in policy and practice settings are lacking [8]. Recent systematic reviews have concluded that there are no valid proxy-report measures of movement behaviours for children under five years of age [9,10].

An important step in the development of self-report tools is understanding whether participants interpret items as intended. One way to assess understanding is through the use of ‘cognitive interviewing’ [11], specifically the think-aloud method. The participant is prompted to narrate their thoughts as they formulate answers to each item, thereby revealing their interpretation and decision-making process. Improving the format and wording of items based on participant feedback can act to decrease the ‘cognitive load’ placed on participants [12] as they complete the questionnaire. This, in turn, may improve an individual’s recall of the behaviour of interest and subsequent accuracy and quality of the data obtained [11].

This paper describes the outcomes from the cognitive interview phase of the development of two brief surveys, the Movement Behaviour Questionnaire-Baby (MBQ-B) and Movement Behaviour Questionnaire-Child (MBQ-C), which are designed to measure the physical activity, screen time, and sleep of infants, toddlers, and pre-schoolers aged 0–5 years. The aims of this development phase were to (1) review the format, content, and clarity of questionnaire items and response options, (2) understand how parents retrieve, encode, and formulate responses when asked about their young child’s movement behaviours, and (3) identify potential sources of response error and make appropriate modifications.

## 2. Materials and Methods

This is a qualitative study. Recruitment, interviews, and iterative analysis occurred between April and August 2020.

### 2.1. Participants and Recruitment

An invitation to participate was distributed via email or text message to a convenience sample of parents in southeast Queensland, Australia, who self-identified as having an infant and/or child 0–5 years of age. If interested, parents were directed to an electronic Participant Information Sheet and Consent form via a Research Electronic Data Capture (REDCap) [13,14] database link. Once consenting, parents were directed to complete a brief demographic survey and then contacted by a research team member via telephone to schedule an interview time.

### 2.2. Movement Behaviour Questionnaire-Baby (MBQ-B) and Movement Behaviour Questionnaire-Child (MBQ-C)

The MBQ-B and MBQ-C are newly developed brief tools designed to measure physical activity, screen time, and sleep in infants, toddlers, or pre-schoolers, up to and including five years of age. Candidate items were identified based on a literature review of existing brief measures [8] and an examination of movement behaviour items used in key Australasian obesity prevention trials [15,16,17,18].

The initial version of the MBQ-B (Supplementary Table S1) contained six items and was designed to be administered to parents of children < 18 months of age who are not yet walking. This version included an item on the frequency and duration of tummy time, assessed time spent in active play/outdoor play, watching television, and using mobile digital devices as single items across a typical day (not separately for weekday and weekend days), and did not include a bedtime routine item. For toddlers and preschool-aged children, the first iteration of the MBQ-C comprised nine items assessing time spent in active play/outdoor play on a typical weekday and weekend day (two items); time spent watching television and using mobile digital devices on a typical weekday and weekend day (four items); time spent in sleep (night-time and during the day); and bedtime routine (three items). For comparison purposes, both open-ended and close-ended response formats were developed and tested.

### 2.3. Cognitive Interview Protocol

The protocols for the cognitive interviews and the standardised interview guide (Supplementary Table S2) were developed using the methodology of Willis [11]. Interviews were conducted via video call by one interviewer (DB) who has extensive experience building rapport with participants as a researcher and health practitioner. The use of video-call allowed flexibility for participants to complete the interview in the comfort of their own home at a time that was convenient to them. There is research to indicate that this can make participants feel more comfortable when disclosing their experiences and that video-call is as effective as in-person interviews [19]. A brief introduction was used to reiterate the purpose of the study and confirm the participant’s consent to video record the interview. This was followed by a ‘warm-up’ question to introduce the think-aloud process. Parents were then asked to review the version of the MBQ relevant to their child’s developmental stage. If the participant had an infant and a child in their household, they were invited to review both the MBQ-B and MBQ-C. The interviewer shared the relevant MBQ version on the screen such that the interviewer and participant could both see the questionnaire.

Two strategies were used to uncover the cognitive processes occurring as parents thought about and developed answers to items: the concurrent think-aloud technique and probing questions. Participants were asked to read the items aloud, think-aloud while formulating an answer to the item, and provide their answer to the interviewer. General probes were used to elicit feedback on the format, content, and clarity of items. For all items, participants were presented with both open-ended and closed-ended response formats and asked to state their preferred format and why. Each interview took approximately 30 min and at the conclusion participants were offered a retail gift card as a ‘thank you’ token to the value of 20AUD.

### 2.4. Data Analysis

Analysis was completed using an iterative process [11]. Upon the completion of each interview, a computer-generated transcription was downloaded and edited for accuracy and completeness against the videorecording (CT). Two members of the research team (ST, RB) independently reviewed each transcript and associated recording, classifying participant responses into categories developed by Tourangeau [20] and further adapted by Willis [11]: General comprehension: was the question understood?; Decision process: was the participant able to articulate a strategy to retrieve information from memory and to arrive at an answer to the question?; Response process: how does the participant map their own answer onto the scale provided?, are the scale responses appropriate?, and preference for open- versus closed-ended options.

At the conclusion of each round of interviews, members of the research team (ST, RB, CT, and DB) discussed participant’s responses. When participants consistently identified problems or items were repeatedly misunderstood, modifications to the questionnaire were proposed and agreed upon, i.e., decisions may have been based on any of the interviews that preceded coding, not necessarily the most recent iteration. The wording of items modified based on the participants’ suggestions was to improve clarity and usability. This process of interviewing, analysis and modification was repeated until the research team reached a consensus that participants showed adequate comprehension of items with no further modifications required. In each subsequent round, the revised questionnaire was tested with new participants or previous participants recontacted if clarification was sought.

## 3. Results

### 3.1. Participants

A total of 29 interviews were conducted with 20 parents between April and August 2020. Nineteen mothers and one father participated, with nine participants aged between 26 and 35 years and the remainder 36–45 years. All except one identified as Caucasian ethnicity and 70% (*n* = 14) had a university degree.

### 3.2. Interviews and Modifications

Four rounds of interviews were completed. Five participants had both an infant and a child within the eligible age range and therefore completed both the MBQ-B and MBQ-C. Table 1, Table 2, Table 3 and Table 4 summarise the main findings related to participant comprehension and processes as well as item modifications at each round. The number of participants interviewed within each round is provided in the footnote of each table. In summary, all six items in the initial version of the MBQ-B underwent minor revisions (e.g., alterations to examples), one item underwent major revisions (milestone associated with tummy time), and one item was added (restrained time). Of the nine items in the initial version of the MBQ-C, one item was retained in its original form (sleep routine), four underwent minor revisions, and four underwent major revisions (weekday and weekend day active play and weekday and weekend day screen time).

## 4. Discussion

The purpose of this paper was to describe the cognitive interview phase of the development of two brief surveys, the Movement Behaviour Questionnaire-Baby (MBQ-B) and Movement Behaviour Questionnaire-Child (MBQ-C), to measure the movement behaviours (physical activity, screen time, and sleep) of children aged 0 to 5 years. There is potential for short tools such as the MBQ to be validated and embedded into nationally representative surveys, offering a feasible and cost-effective way to monitor health behaviours of the population over time. Given the opportunity for these surveys to inform public health policy and practice, it is essential that steps are taken to enhance the accuracy of the behaviours being assessed.

Research participants move through a complex set of processes while answering questionnaire items, including understanding the question, recalling the relevant behaviour, inference and estimation, mapping their answer onto the response format, and, finally, editing the answer for social desirability [21]. The first aim of this research was to use cognitive interviews with participants to review—and ultimately improve—the format, content, and clarity of questionnaire items and response options. General comprehension was high, overall, questions were well understood, and most items required only minor revisions.

The second aim was to understand how parents retrieve, encode, and formulate responses when asked about their young child’s movement behaviours, and participants were able to articulate their decision process during the interviews. Most often, they recalled their usual family routines and rules when estimating the duration and/or frequency of behaviours, especially for time spent in active play and use of electronic devices. To estimate the duration of outside play, parents referred to the child’s daily routine, considering wake and bedtimes, daytime naps, or eating occasions. However, this process highlighted a potential source of response error (aim 3) in that it became apparent that any outside time was equated as being active play, regardless of the actual intensity of activity that children may engage in outdoors. This problem was compounded by the use of examples within items. When recalling the duration of play, participants were unable to infer beyond the specific examples provided, taking the list of items literally, rather than being an indication of the level of intensity of the movement behaviours of interest. The phenomenon of interpreting items literally has been observed in other studies that utilised cognitive interviewing [22].

As a result, modifications were made to items and associated examples to improve understanding and recall using a technique known as ‘decomposing’ [21]. Decomposing the general item about outdoor play from iteration 1 into more specific questions—with examples of active play (walking, running, dancing, climbing, playing with balls, riding bikes or scooters, swimming) and within this, vigorous play (running, jumping, dancing, riding bikes or scooters)—during iteration 2 was well-received by participants. This may be because decomposing a general question into several more specific ones is useful when the specific questions relate to less frequent or memorable behaviours [21].

While decomposition can improve the accuracy of recall for less frequent behaviours [23] (for example, sedentary time decomposed to highlight time spent restrained in a high chair or car seat), this technique may be less useful when asking about common, repeated behaviours. Participants did report having difficulty accurately recalling active play and tummy time with infants, and considered these as activities of high frequency, spontaneous, and of short duration. For frequently occurring behaviours, parents or caregivers may never encode the relevant information in the first place, and are therefore unable to recall the relevant behaviours when prompted [23].

The use of judicious examples was also relevant when assessing the use of screen-based devices. It is challenging to develop brief items that adequately capture the numerous and often times simultaneous screen-based activities that infants and children might engage in [24]. Participants mentioned the need to include video calls as an example within items. This is unsurprising given data collection occurred during the COVID-19 pandemic, when restrictions to movement between states and countries, and even within individual cities, necessitated a more frequent use of video calls to connect with friends and family.

Participants noted that routines differ on weekdays compared with weekends, and easily differentiated between the two. This has also been reported during cognitive interviews with Korean American families with children aged 2–5 years, with subsequent differences in screen time duration reported [25], which highlights the need to include items that differentiate between the weekday and weekend. While this may increase the overall number of items, participant burden should remain low. Employing techniques such as cognitive interviewing to refine questionnaire items can act to decrease the ‘cognitive load’ placed on participants during survey completion and ultimately improves the accuracy of recall.

Schwarz and Oyserman [21] describe the “recall and count” strategy, i.e., “researchers typically hope that respondents will identify the behaviour of interest, scan the reference period, retrieve all instances that match the target behaviour, and finally count these instances to determine the overall frequency of the behaviour”, highlighting the need for numeracy skills to support accurate reporting. This was evident within this sample when participants reported calculating the difference between bedtime and waketime to estimate sleep duration or multiplying the duration of a favourite television show by number of episodes watched per day to determine a duration for the use of a screen-based device.

When considering the response process, there was no clear preference for open versus closed responses. Both formats have advantages and disadvantages. An open-ended response option ‘forces’ parents to think specifically about their own child’s behaviour rather than choose from a list available. Closed-ended options might help put an item in context for the participant, but might also prompt a participant to edit their answer for social desirability [23], such as when the middle option is perceived as ‘typical’ or ideal behaviour. Both open- and closed-ended response options will be taken forward to the MBQ validation study to assess which options result in greater accuracy of parental reporting of children’s movement behaviours.

### Strengths and Limitations

A strength of this research is the use of multiple rounds of interviews in which items could be modified by the research team and retested with participants. There was a higher proportion of participants with a tertiary education compared to the national average.; seventy percent in this study, compared with 50% of Australian women aged 25–44 years having a qualification at bachelor’s degree level or above in 2022 [26]. A sample with lower literacy and numeracy skills or with English as a second language may provide different feedback on comprehension and wording of items.

## 5. Conclusions

Cognitive interviews and iterative coding rounds addressing the format, content, and clarity of the questionnaire items and response options informed revisions to item wording, judicious use of examples, and recall prompts in the MBQ-B and MBQ-C. These versions will be taken forward into validation studies evaluating the test–retest reliability and concurrent validity of the items. Cognitive interviewing enhanced our understanding of how parents retrieve, encode, and formulate responses to questions about their young child’s movement behaviours, increasing our confidence that questionnaire items are correctly interpreted and understood by participants.

## Figures and Tables

**Table 1:** Main findings and modifications to the Movement Behaviour Questionnaire-Baby (MBQ-B) and Movement Behaviour Questionnaire-Child (MBQ-C) after first round of interviews.

MBQ-B *		
Item	Main findings	Modifications and actions
**1a** Thinking about the past week, how many times EACH DAY did you usually place your infant on their tummy for play (tummy time on their stomach while awake)? **1b** How long did each “tummy time” usually last?	General comprehension: Respondent questioned the applicability of this item for children aged 12 months or older age: “I know you’re asking for… it’s just that this is hard because he’s not this little, so technically this doesn’t apply… he’s one and he, we don’t do tummy time”.	Nil at this stage; decision made by research team continue with interviews before making changes to this item
**2a** Thinking about the past week, how many times EACH DAY did you usually do some active play with your infant? Active play could be lying on the floor with your infant on your legs and lifting, dancing with your infant, flying and lowering your infant so they are upside down. **2b** How long did each “active play time” usually last?	General comprehension: Examples of active play considered unclear “lying on the floor with your infant on your legs and lifting... (participant reads again) lying on the floor with your infant on your legs and lifting... Okay, that’s a little bit unclear”.	Examples of active play revised: Active play could be lying on the floor with your infant on your legs, lifting, dancing with your infant, or playing action games like pat-a-cake or peek-a-boo.
**3** Thinking about the past week, on a typical day (24 hours) how much time did your infant spend watching television programs, videos/internet clips or movies on a television, computer or portable/mobile device such as tablet or smartphone? (e.g., 2 h 15 min)	General comprehension: Inclusion of (24 h) was not considered helpful or necessary “I think it’s pretty straightforward. I wouldn’t necessarily need the 24 hours. I would just assume that it’s for the full day”. Decision process: Draw on family rules related to devices when considering time on each device. E.g., Participant quickly came to a response of zero “my one-year-old doesn’t really, he doesn’t do screen time at all, but he’s one. Ask me about my five-year-old! [laughing]”.	Redundancy of 24 h noted, but nil changes at this stage; continue with interviews
**4** Thinking about the past week, on a typical day (24 h) how much time did your infant spend playing games or using apps on electronic devices such as a computer or laptop, videogame console, iPad, tablet, smartphone, or any electronic gaming device? (e.g., 2 h 15 min)	General comprehension: well understood with nil concerns noted.	Nil modifications; continue with interviews
**5** Thinking about your infant’s sleep during the past week, how much time did your infant spend in sleep during the NIGHT (between 7 in the evening and 7 in the morning)? (e.g., 2 h 15 min)	General comprehension: The phrase ‘spend in sleep’ was unclear “That’s a bit funny there… how much time did your infant spend in sleep during the night?” Response process: Participant preferred closed ended responses to open “it was easy just to see it there and select something that might be appropriate, as opposed to overthinking and calculating”	Concerns with wording noted, but nil modifications at this stage; continue with interviews
**6** Thinking about your infant’s sleep during the past week, how much time did your infant spend in sleep during the DAY (between 7 in the morning and 7 in the evening)? (e.g., 2 h 15 min)	General comprehension: As above “it’s just unnatural for me to say that... How much time did you spend in sleep?”	Concerns with wording noted, but nil modifications at this stage; continue with interviews
**MBQ-C** *		
**Item**	**Main findings**	**Modifications and actions**
**1** Thinking about the past week, on a typical weekday (24 h) how much time did your child spend playing outdoors?	General comprehension: “I didn’t feel the need for the 24 h” Decision process: Participants recalled usual family routine to determine time spent outside “Well just given his awake time… he sleeps for two hours in the middle of the day. And then we’ve got the morning and afternoon are two sessions and then you’d have to average it out so”. “Our days are sort of broken up a little bit into chunks… before (younger sibling’s) nap, and then when he’s napping, and then when he’s up. So, I guess... set chunks of time” Response process: Parents reporting **any time** spent outside—item not capturing time spent in active play or vigorous activities, could lead to overestimate of time in play.	Item revised to specify ‘active play’ + examples. Sub-item about vigorous play added. **1a** Thinking about the past week, on a typical weekday (24 h) how much time did your child spend in active play outdoors, which includes walking, running, dancing, climbing, playing with balls, riding bikes or scooters, swimming? **1b** Of this time, how much was spent doing vigorous activities such as running, jumping, dancing, riding bikes or scooters? Item tested in iteration 2
**2** Thinking about the past week, on a typical weekend day (24 h) how much time did your child spend playing outdoors?	General comprehension: Participants generally reported there was more time for physical activity on the weekend—weekdays are constrained by work commitments, “our weekends are more free to spend time with (child)”; “the kid’s dad is home and he’s super active, so usually we’d be spending time outside”. Decision process: Similar cognitive processes for weekend day as weekday—referring to usual routine “Again, I think my strategy like the weekdays would be just really breaking up into chunks of time”. But lack of work-related routine sometimes made it more difficult to estimates time spent outdoors “This is a little bit trickier because... it’s a bit more fluctuating with what we’ll do on the weekend. But generally, I’d say probably similar to during the week, maybe a little bit more”. Response process: Participants varied in their preference for open versus closed responses. While some felt it improved accuracy by having choices available, others felt that parents would choose quickly and without much thought, thereby decreasing accuracy. “I tend to use multiple choice answers as a process of elimination. Not generally trying to find the answer, trying to eliminate the wrong ones. And with these questions where I do know the answer, for me, I just find worded answers far easier to come to, than sifting through multiple choice questions”.	Same modifications made as per weekday item
**3** Thinking about the past week, on a typical weekday (24 h) how much time did your child spend watching television programs, videos/internet clips or movies on a television, computer or portable/mobile device such as tablet or smartphone?	General comprehension: No specific concerns noted “(it) takes a bit of brain power... but I think everything is quite clear there if you read through the question thoroughly”. Decision process: Participants think about their child’s routine, as well as family rules about access to devices, to determine total time spent “I would be starting from the morning and thinking through the two times we let him watch screen time, and I’d be able to easily come to an answer”. “Because our daughter who’s three, doesn’t do any form of the technology outside of the TV”. Decision process: Participant wonders how to calculate an answer when child does not have screen time most days. She defaults to calculating an average rather than describing a ‘typical’ day—when interviewer asks “is the question difficult to answer?” participant responds “Ah, well only because she doesn’t watch it, every day. Then you’ve got to average out… how many minutes on days where some days are zero?”	Nil at this stage; continue with interviews; research team to consider how participants interpret ‘typical’ in subsequent rounds.
**4** Thinking about the past week, on a typical weekend day (24 h) how much time did your child spend watching television programs, videos/internet clips or movies on a television, computer or portable/mobile device such as tablet or smartphone?	Response process: preference for open vs. closed response options varied for this item also. For one participant that preferred an open-ended option earlier, now preferred closed. “I don’t know, it’s kind of the opposite of what I said to the physical activity (question), but, um, I guess I find it easier to quickly come to an answer... But, then again, I might be more confident that I had a more accurate answer possibly…”	Nil at this stage; continue with interviews
**5** Thinking about the past week, on a typical weekday (24 h), how much time did your child spend playing games or using apps on electronic devices such as a computer or laptop, videogame console, iPad, tablet, smartphone, or any electronic gaming device?	Decision Process: Participants referred to their household rules and limits regarding these activities. Most children were not allowed to play games and participants could quickly and confidently answer ‘zero’, “We try not to let him on the computer or have an iPad or anything like that”.	Nil; continue with interviews
**6** Thinking about the past week, on a typical weekend day (24 h), how much time did your child spend playing games or using apps on electronic devices such as a computer or laptop, videogame console, iPad, tablet, smartphone, or any electronic gaming device?	Decision Process: As above, participants referred to their household rules and limits regarding these activities. Lack of work-related routine on weekends.	Nil; continue with interviews
**7** Thinking about the past week, how much time did your child spend in sleep during the NIGHT? (e.g., 2 h 15 min)	Decision Process: Numeracy skills required—participants refer to child’s bedtime, then waketime and calculate the difference: “she goes down at about seven and wakes up at about five. So maybe about 10 h”.	Nil; continue with interviews
**8** Thinking about the past week, how much time did your child spend in sleep during the DAY? (e.g., 2 h 15 min)	Decision Process: Participants could come to an answer quickly because they monitor their child’s sleep. Adequacy of sleep was a commonly reported concern of parents: “I tend to be fairly aware of their sleep”.	Nil; continue with interviews
**9** In a typical week, how often does your child have a regular bedtime routine (e.g., bath, story)? e.g., 5 nights	Decision Process: All participants answered this quickly, with no apparent difficulties in interpretation.	Nil; continue with interviews

* Six interviews—five participants completed the MBQ-C, and one participant completed both versions.

**Table 2:** Main findings and modifications to the Movement Behaviour Questionnaire-Baby (MBQ-B) and Movement Behaviour Questionnaire-Child (MBQ-C) after second round of interviews.

MBQ-B *		
Item	Main findings	Modifications and actions
**1a** Thinking about the past week, how many times EACH DAY did you usually place your infant on their tummy for play (tummy time on their stomach while awake)? **1b** How long did each “tummy time” usually last?	General comprehension: The two participants questioned the relevance of this item to infants who can crawl: “it’s a little bit difficult, this one, because he’s on his tummy a lot, but at the same time, he’s very mobile... when he was pretty little, he couldn’t crawl, I would put him (down for) tummy time”	Nil at this stage; decision made by research team continue with interviews before making changes to this item
**MBQ-C** *		
**Item**	**Main findings**	**Modifications and actions**
**1a** Thinking about the past week, on a typical weekday (24 h) how much time did your child spend in active play outdoors, which includes walking, running, dancing, climbing, playing with balls, riding bikes or scooters, swimming? **1b** Of this time, how much was spent doing vigorous activities such as running, jumping, dancing, riding bikes or scooters?	General comprehension: Addition of examples and sub item was well received. Decision process: Sub item successfully prompted participants to recall activities of different intensities. “Of this time, how much was spent doing vigorous activities? I would say like 80 to 90% of that… she doesn’t stop… except there’s sometimes she likes to sit and play with rocks. So, but that usually doesn’t last for too long. So, I just minus that particular activity off”. Explanation of decision process revealing numeracy skills required “So, of this time, how much time is spent doing vigorous activity? I’d say probably one hour of that in a day… It’s around about a half an hour or so that she would be doing scootering in the afternoon… And then she just does little bursts of play, where they’ll play tag for 10 min or hide and seek where they’re running around the backyard”.	Nil; continue with interviews
**2a** Thinking about the past week, on a typical weekend day (24 h) how much time did your child spend in active play outdoors, which includes walking, running, dancing, climbing, playing with balls, riding bikes or scooters, swimming? **2b** Of this time, how much was spent doing vigorous activities such as running, jumping, dancing, riding bikes or scooters?	General comprehension: Addition of sub item was well received	Nil; continue with interviews
**3** Thinking about the past week, on a typical weekday (24 h) how much time did your child spend watching television programs, videos/internet clips or movies on a television, computer or portable/mobile device such as tablet or smartphone?	General comprehension: participants understood that the intent of the items was to capture time when the child is not moving. This resulted in them thinking about whether children were moving between sitting and standing “I would question… wonder whether... I guess she does a bit standing up… um… Looking at those devices, so I’d wonder whether that counts or not”.	Addition of ‘standing time’ sub item 3b Of this time, how much time did they watch an electronic device while standing? (e.g., 0 h 30 min), with a warning that the time provided must be less than the previous answer.
**4** Thinking about the past week, on a typical weekend day (24 h) how much time did your child spend watching television programs, videos/internet clips or movies on a television, computer, or portable/mobile device such as tablet or smartphone?	Decision process: Participants referring to their altered routine on the weekend “Zero time on the weekend. They tend to not remember even about the iPad and... they don’t play on our phones, but because we’re all together. They don’t tend to not use them on the weekends.”	As above, addition of ‘standing time’ sub item.

* Six interviews with new participants—four completed MBQ-C and two completed both versions.

**Table 3:** Main findings and modifications to the Movement Behaviour Questionnaire-Baby (MBQ-B) and Movement Behaviour Questionnaire-Child (MBQ-C) after third round of interviews.

MBQ-B *		
Item	Main findings	Modifications and actions
**1a** Thinking about the past week, how many times EACH DAY did you usually place your infant on their tummy for play (tummy time on their stomach while awake)? **1b** How long did each “tummy time” usually last?	General comprehension: Further querying of whether item applicable now that infant is crawling. “He is... already passed that time that I need to, um…actively or proactively put him on tummy... these tummy time questions don’t really apply to my baby anymore because he can actually crawl, really, really fast”. “he’s um, sitting and crawling and everything... he doesn’t need tummy time anymore. He wouldn’t stay there even if I tried to”. General comprehension: No clear preference for the term ‘baby’ versus ‘infant’. Response process: Tummy time was challenging to recall because it was done frequently throughout the day but briefly. “I would have to calculate it… too hard! … yeah, I’d have to calculate it and sort of add up every time she’s on the ground. So, she’s not walking yet, so she’ll sit up and then she’ll roll, you know, onto her tummy and play. So, I mean, at a guess it’s probably not going to be that accurate… oh my god, I don’t know how to answer that question.”	Branching logic added to software: Does your baby crawl (yes/no)? If the baby has reached their ‘crawling’ milestone, parents were directed to answer item; if not, this item was deemed ‘not applicable’ and parents –skipped this item and were directed to next item. Terminology revised to ‘baby’ across all items in the infant version to enhance readability of questionnaire. Daily frequency replaced with weekly frequency. Item as well as open- and closed-ended responses revised. 1. This question is about the times when your baby is awake and placed on their tummy for playtime while you are watching them. Thinking about the past week, on how many days did you place your baby on their tummy for play?
**2a** Thinking about the past week, how many times EACH DAY did you usually do some active play with your infant? Active play could be laying on the floor with your infant on your legs, lifting, dancing with your infant, or playing action games like pat-a-cake or peek-a-boo. **2b** How long did each “active play time” usually last?	Decision process: Participants struggling with recall due to the short duration but high frequency of these activities “Again, it’s difficult to say a set amount of times, because you do a lot of these things without even realizing you’re doing it” “When we do something like that, it is quick. It’s me just kind of picking him up and quickly having a little game while we’re moving from one place to another... rather than, you know, it being a kind of deliberate, sit down, um, ‘let’s play a game Alex’. It’s... something that spontaneously happens every now and then, throughout a day”	Daily frequency and duration changed to total duration on typical day. Examples revised: 2 Thinking about the past week, on a TYPICAL DAY, how much time in total did you do some active play with your baby? Active play can be playing with toys or objects while lying or sitting on the floor, crawling on the floor or through tunnels, or pulling up to a standing position while holding on to furniture.
**3** Thinking about the past week, on a typical day (24 h) how much time did your infant spend watching television programs, videos/internet clips or movies on a television, computer, or portable/mobile device such as tablet or smartphone? (e.g., 2 h 15 min)	General comprehension: consistently reporting that ’24 h’ not required. Decision process: Refer to usual routine “it tends to be a very specific, like an episode of a TV show or something like that, where it runs for a certain amount of time and you tend to know how long it runs for and maybe you only let your kids watch like, one episode of something or two”? Decision Process: Concern that example “(e.g., 2 h 15 min)” could introduce social desirability bias if parents perceive this as the ‘acceptable’ amount of time.	‘24 h’ and example response removed. 3 Thinking about the past week, on a TYPICAL DAY, how much time did your baby spend watching television programs, videos/internet clips or movies on a television, computer, or portable/mobile device such as iPad, tablet or smartphone?
**4** Thinking about the past week, on a typical day (24 h) how much time did your infant spend playing games or using apps on electronic devices such as a computer or laptop, videogame console, iPad, tablet, smartphone, or any electronic gaming device? (e.g., 2 h 15 min)	Decision process: Participants felt infants were too young to play with games or apps, and quickly came to a response of zero, but were unsure whether to include video chatting, “the thing that’s excluded is FaceTime, when you’re face timing relatives or friends... do you want people to count that?” Indicates that examples are not capturing how infants interact with screens. Response process: preference for open vs. closed responses remains mixed across participants.“I preferred being able to put in the answer that was exactly what I wanted to say, rather than having to choose an answer option that was close, but not quite correct”.	‘Playing games or using apps’ changed to ‘looking at photos, or video chatting (e.g., FaceTime, Zoom, Skype) on a screen-based device’. As for previous item, ‘24 h’ and example times removed. 4 Thinking about the past week, on a TYPICAL DAY, how much time did your baby spend playing games, looking at photos, or video chatting (e.g., FaceTime, Zoom, Skype) on a screen-based device such as a computer or laptop, video game console, iPad, tablet, or smartphone?
**5** Thinking about your infant’s sleep during the past week, how much time did your infant spend in sleep during the NIGHT (between 7 in the evening and 7 in the morning)? (e.g., 2 h 15 min)	General comprehension: Defining day and night was not needed or helpful—participants understood what these meant. General comprehension: The phrase ‘in sleep’ was intended to prompt participants to calculate the time their infant was asleep minus wakefulness, however, it continued to create confusion during this round.	‘Spend in sleep’ changed to ‘sleep in total’; addition of ‘on a typical night’; definition of night removed, and example times removed. 5 Thinking about the past week, on a typical night, how many hours/minutes did your baby sleep in total during the night?
**6** Thinking about your infant’s sleep during the past week, how much time did your infant spend in sleep during the DAY (between 7 in the morning and 7 in the evening)? (e.g., 2 h 15 min)	General comprehension as per item 5.	‘Spend in sleep’ changed to ‘sleep in total’; addition of ‘on a typical day’, definition of day and example times removed. 6 Thinking about the past week, on a typical day, how many hours/minutes did your baby sleep in total during the day?
**MBQ-C** *		
**Item**	**Main findings**	**Modifications, actions, and item taken forward to validation study**
**1a** Thinking about the past week, on a typical weekday (24 h) how much time did your child spend in active play outdoors, which includes walking, running, dancing, climbing, playing with balls, riding bikes or scooters, swimming? **1b** Of this time, how much was spent doing vigorous activities such as running, jumping, dancing, riding bikes or scooters?	General Comprehension: Participants found examples useful as prompts to distinguish between active play and play that is sedentary “often the girls will come out in the morning around morning-teatime, and they’ll play outside... they’ll often do fairly active games... but one of the things that they do (is) potter outside... playing in their cubby house and in the sand pit, which are not quite as active... Those weren’t included (in the examples so) I’d probably maybe reduce that to two hours”. General question: Question excludes time spent in active play indoors. Parents understood this, but item may not capture all active play “The weather’s quite cold and not always conducive with outdoor play... we still try and get out there... on average, I would say about two hours outside on a weekday”. Decision Process: Most parents did not read ’24 h’ out loud and reported use of ‘(24 h)’ as redundant “I don’t really know of any children that are playing outside overnight anyway”.	Given participants clearly articulated their thought process i.e., calculate how many hours the child was awake, then take way time spent in other activities to calculate active play, this demonstrated they are considering the full 24 h period, including activities after dark. Therefore ‘(24 h)’ and ‘outdoor’ removed. 1a Thinking about the past week, on a TYPICAL WEEKDAY, how much time did your child spend in active play? Active play includes activities such as walking, running, dancing, climbing, playing with balls, riding bikes or scooters, or swimming. 1b Of this time, how much was spent doing vigorous activities such as running, jumping, dancing, riding bikes or scooters?
**3a** Thinking about the past week, on a typical weekday (24 h) how much time did your child spend watching television programs, videos/internet clips or movies on a television, computer, or portable/mobile device such as tablet or smartphone? **3b** Of this time, how much time did they watch an electronic device while standing?	General Comprehension: Addition of sub-item about standing was well received; however, ‘electronic device’ did not prompt participants to think about television. “...an electronic device, I straightaway think of a tablet or smartphone something portable. So, my head... didn’t automatically go to thinking about television”. Another participant initially said zero in response to sub-item, for the same reason “That’s why I said zero, but if we’re talking about the actual TV while standing. James is a bit of a mover and a groover, so he will stand up, he’ll jump on the couch, he’ll lie down. So, he might be standing for a small portion of that time... maybe 30 min... he’s standing during that two-hour period”. Decision Process: Participants were confident of their child’s preference to either sit or stand, which resulted in a quick and confident response to the sub-item. “It could be sitting playing with toys in front of the TV. It’s... not just sitting on the couch, but he’s rarely up and about when something’s on”. Response process: Closed screen time response options required additional choices to accommodate higher screen time estimates “He’s very much into TV. Basically, so he could watch that hour in the morning, possibly an hour in the afternoon, and then even a movie in the evening with us or with his brother or something like that. So, it could be to my disgust about four hours on a weekend.” Suggested modifications to wording: Participants preferred questions related to standing as opposed to “... how much time did they watch and electronic device while sitting”. Asking about the exception to the rule made sense to participants.	‘(24 h)’ removed. Sub-item revised—‘electronic device’ replaced with television programs, videos/internet clips, or movies. Additional closed response options added, Between 2 and 3 h per day’ and ‘More than 3 h per day’. 3a Thinking about the past week, on a typical weekday, how much time did your child spend watching television programs, videos/internet clips or movies on a television, computer, or portable/mobile device such as iPad, tablet or smartphone? 3b Of this time, how much time did they spend watching television programs, videos/internet clips, or movies while standing?
**4a** Thinking about the past week, on a typical weekend day (24 h) how much time did your child spend watching television programs, videos/internet clips or movies on a television, computer, or portable/mobile device such as tablet or smartphone? **4b** Of this time, how much time did they watch an electronic device while standing?	Suggested modifications to wording: Participants reported that the types of screens asked about in the question were adequate. Could not provide additional examples to add.	Modifications made as per weekday items 3a and 3b
**5a** Thinking about the past week, on a typical weekday (24 h), how much time did your child spend playing games or using apps on electronic devices such as a computer or laptop, videogame console, iPad, tablet, smartphone, or any electronic gaming device? **5b** Of this time, how much time did they play with an electronic device while standing?	Decision Process: Some participants were unsure of whether to include screen-based communication like FaceTime or to include the use of electronic toys, e.g., imitation laptops with musical buttons “he’s got some toys that are electronic, that make noise and things, but I probably wouldn’t put it in that same category that you’re after”. Response Process: As with 3a and 3b, parents indicated closed screen time response options required additional choices to accommodate higher screen time estimates.	‘(24 h)’ removed. ‘Electronic device’ replaced with screen-based device and examples revised to include looking at photos and video chat. Two more closed options added to responses: - Between 2 and 3 h per day - More than 3 h per day 5a Thinking about the past week, on a typical weekday, how much time did your child spend playing games, looking at photos, or video chatting (e.g., FaceTime, Zoom, Skype) on a screen-based device such as a computer or laptop, video game console, iPad, tablet, or smartphone? 5b Of this time, how much time did they spend playing games, looking at photos, or video chatting (e.g., FaceTime, Zoom, Skype) while standing?
**6a** Thinking about the past week, on a typical weekend day (24 h), how much time did your child spend playing games or using apps on electronic devices such as a computer or laptop, videogame console, iPad, tablet, smartphone, or any electronic gaming device? **6b** Of this time, how much time did they play with an electronic device while standing?	Decision process for these items consistent with 5a and 5b.	6a Thinking about the past week, on a typical weekend day, how much time did your child spend playing games, looking at photos, or video chatting (e.g., FaceTime, Zoom, Skype) on a screen-based device such as a computer or laptop, video game console, iPad, tablet, or smartphone? 6b Of this time, how much time did they spend playing games, looking at photos, or video chatting (e.g., FaceTime, Zoom, Skype) while standing?
**7** Thinking about the past week, how much time did your child spend in sleep during the NIGHT? (e.g., 2 h 15 min)	General comprehension: The example response is not relevant to night-time sleep “(I need to) come up with a number that reflects all seven nights, and then the number at the end is throwing me because it says two hours, you know, for example two hours and 15 min”. The phrase ‘spend in sleep’ continued to create confusion. Decision Process: Consistent with previous rounds, participants referred to the child’s bedtime, then waketime and calculated the difference “he goes to bed normally about 7.30 and then he sleeps until most days until 6.30. So, um, I would say, what’s that? I’m just trying to work that out, maybe between 10- and 11-h sleep”. Response process: Some participants interpreted the question as asking about the total amount of sleep over the week i.e., adding up seven nights worth of sleep, rather than a typical night “if we’re talking about during the night, each night he sleeps for about nine or 10 h, and if it’s talking about the last week, am I giving a weekly amount?” Having closed responses aided with the interpretation of the question—the range of options available made it clearer that the question was asking for an estimate for one night, not the total for the week “it’s a lot easier to give an answer, having those options to choose from”.	’Spend in sleep’ changed to ‘sleep in total’, ‘on a typical night’ added, and example times removed. 7 Thinking about the past week, on a TYPICAL NIGHT, how many hours/minutes did your child sleep in total during the night?
**8** Thinking about the past week, how much time did your child spend in sleep during the DAY? (e.g., 2 h 15 min)	General comprehension: The phrase ‘spend in sleep’ continued to create confusion.	‘Spend in sleep’ changed to ‘sleep in total’, ‘on a typical day’ added, and example times removed to avoid social desirability bias in reporting, e.g., if participants perceive this is an ideal sleep duration, they may alter their response accordingly. 8 Thinking about the past week, on a TYPICAL DAY, how many hours/minutes did your child sleep in total during the day?

* Nine interviews—MBQ-C completed five times and MBQ-B five times. All were new participants, except one, who had completed the MBQ-C in round one and subsequently completed the MBQ-B.

**Table 4:** Main findings and modifications to the Movement Behaviour Questionnaire-Baby (MBQ-B) after fourth round of interviews.

MBQ-B *		
Item	Main findings	Modifications, actions and item taken forward to validation study
**1** This question is about the times when your baby is awake and placed on their tummy for playtime while you are watching them. Thinking about the past week, on how many days did you place your baby on their tummy for play?	General comprehension: Participants noting that tummy time is less relevant once infant is rolling. Response process: Requirement to recall over the duration of a week resulting in ceiling effect	Branching logic about crawling which was added to software after third round of interviews modified to: Does your baby roll? Yes/No If the baby has reached their ‘rolling’ milestone, parents were directed to answer item; if not, this item was deemed ‘not applicable’ and parents skipped this item and were directed to next item. Responses revert to daily frequency. 1 Thinking about the past week, how many times EACH DAY did you usually place your baby on their tummy for play?
**2** Thinking about the past week, on a TYPICAL DAY, how much time in total did you do some active play with your baby? Active play can be playing with toys or objects while lying or sitting on the floor, crawling on the floor or through tunnels, or pulling up to a standing position while holding on to furniture.	General comprehension: Participant consistently misinterpreted the definition of active play as reflected in their decision-making process. “it has made me think maybe what I considered active play, isn’t what was being asked” “it depends on how the mums’ interpret active play” Decision process: Participants recalled their daily routine to come to an answer, and systematically recalled active play as all time that their child was not sleeping or eating. “I tend to work backwards... taking away that overnight sleep time, to starting like a whole day. Um, hours per day, and then I’m taking off... naptime and then other sedentary time, where they’re not doing anything (like in the car)” Some participants also included time spent in highchairs and prams depending on what the child was doing “so if he’s sitting in the pram or car but is playing with a toy, that could be counted here?” “24 h, minus the 11 h sleeping, then it will be 13 plus nap time, it will be 11, plus eating, maybe nine to 10 h... if he’s awake and not eating then he would be like, doing all this (referring to examples in question)” “He’s just active, just go all the time”. Response Process: Due to the misinterpretation of the definition of active play, several participants overestimated time when presented with the open option. For example, one participant chose seven hours for an open response, and was then surprised to see the upper limit of closed responses offered was ‘more than 2 h per day’, “I’m a little surprised that, that more than two hours per day is maybe the top, the highest answer option”. Another, when asked if the response she wanted to provide was there, replied “Um, I guess technically no, but five hours is more than two hours per day, so, yes.”	Examples revised to be consistent with Australian physical activity and sedentary behaviour guidelines which emphasis supervised interactive play. 2 Thinking about the past week, on a TYPICAL DAY, how much time in total did you do some active play with your baby? Active play could be crawling on the floor with your baby, rolling around the floor with your baby, playing at the park, dancing with your baby, chasing your baby. Item related to restraint time added. 3 Thinking about the past week, on a TYPICAL DAY, how much time in total did your baby spend in a baby carrier or sling, car seat or capsule, stroller or pram, highchair, bouncer, jolly jumper or play pen?
**3** Thinking about the past week, on a TYPICAL DAY, how much time did your baby spend watching television programs, videos/internet clips or movies on a television, computer or portable/mobile device such as iPad, tablet or smartphone?	Decision process: Participants refer to usual routine, or the length of their child’s favourite television show to derive an answer “I try to limit him, like maybe 15 min in the morning, when I’m trying to get ready... I get him changed, and he still wants me, he still wants to cling on to me. Then I just put him in front of the TV and just play that show so I can get myself ready... give him like 10 to 15 min, play a few songs and yeah”. Response Process: preference for open versus closed responses varied. “I feel like I can give a precise response there, rather than giving a range where I’m somewhere within that range... I was like, being precise so, I like the (open response)”. “I think for timewise (it’s) always easier to be given a range to pick from because... it’s harder to figure out a definite answer for time”.	Nil modifications. Item taken forward to validation study as is.
**4** Thinking about the past week, on a TYPICAL DAY, how much time did your baby spend playing games, looking at photos, or video chatting (e.g., FaceTime, Zoom, Skype) on a screen-based device such as a computer or laptop, video game console, iPad, tablet, or smartphone?	General comprehension: Participants understood the term ‘video chatting’ and examples “I think they’re good examples”. Decision process: Participants found this more challenging to quantify than the previous question as watching television tends to happen daily, while these activities are not “this one’s probably less regular than just the TV, um, because he, he’s just still so little”. For these activities which happened infrequently, e.g., once per week, participants proceeded to mentally average the time over seven days, rather than consider what might happen on a ‘typical day’. “I can’t give an answer there because (we) don’t video chat with… anyone every single day. Maybe video chat once a week with my parents because they’re overseas and with my husband’s parents for around 10 to 15 min... Because I don’t do it every day... I can’t give an average time that my son (would) do it every day, on a typical day. Participant: “Five minutes” DB: “How do you come to that answer?” Participant: “Average out the time spent... if he’s on 30 min each week. Then I average it out to seven days”	Nil modifications to item, but definition of a ‘typical day’ added to instructions for the questionnaire as a whole “A typical day is something your child does on most days”.
**5** Thinking about the past week, on a TYPICAL NIGHT, how much time did your baby sleep in total during the night?	Nil concerns noted in this round.	Nil modifications. Item taken forward to validation study as is.
**6** Thinking about the past week, on a TYPICAL DAY, how much time did your baby sleep in total during the day?	Response Process: In this round, participants reported a preference for closed response options which provide a range, rather than having to decide on fixed number for the open response “It’s hard to have a definite, definite answer for time, because we’re human, we don’t... have a strict schedule but it’s hard to actually have like a definite answer for time, because it can be different every day. That’s why I think having a range is easier”.	Nil modifications.

* Eight interviews—all previous participants who were parents of infants.

## Data Availability

Please contact the corresponding author about the availability of data.

## Supplementary Materials

The following supporting information can be downloaded at: https://www.mdpi.com/article/10.3390/children10091554/s1, Table S1: Iterations of the Movement Behaviour Questionnaire-Baby (MBQ-B) and Movement Behaviour Questionnaire-Child (MBQ-C) tested in the first round of cognitive interviews, showing open and closed response options; Table S2: Interview outline.
