# Supplementary material for: Cognitive Testing of Items Measuring Movement Behaviours in Young Children Aged Zero to Five Years: Development of the Movement Behaviour Questionnaires for -Baby (MBQ-B) and -Child (MBQ-C)
Source: Children (Basel). 2023 Sep 14;10(9):1554. doi: 10.3390/children10091554 (PMC10529515; doi:10.3390/children10091554)
Supplement: Supplementary file 1 [file children-10-01554-s001.zip › children-2572416-supplementary.pdf]

Table S1. Iterations of the Movement Behaviour Questionnaire-Baby (MBQ-B) and Movement Behaviour Questionnaire-Child (MBQ-C) tested in the first round of cognitive interviews, showing open and closed response options.

	**MBQ -B**		
**Item no.**	**Item**	**Open response**	**Closed response options**
**1a**	Thinking about the past week, how many times EACH DAY did you usually place your infant on their tummy for play (tummy time on their stomach while awake)?	Number of times	0 – 10
**1b**	How long did each "tummy time" usually last?	Minutes	Less than 5 min; 6 - 10 min; 11 - 15 min; 16 - 20 min; 21 - 25 min; 26 - 30 min; More than 30 min
**2a**	Thinking about the past week, how many times EACH DAY did you usually do some active play with your infant?<br>*Active play could be lying on the floor with your infant on your legs and lifting, dancing with your infant, flying and lowering your infant so they are upside down*	Number of times	0 – 10
**2b**	How long did each "active play time" usually last?	Minutes	Less than 5 min; 6 - 10 min; 11 - 15 min; 16 - 20 min; 21 - 25 min; 26 - 30 min; More than 30 min

**3**	Thinking about the past week, on a typical day (24 hrs) how much time did your infant spend watching television programs, videos/internet clips or movies on a television, computer or portable/mobile device such as tablet or smartphone? (e.g., 2 hours 15 minutes)	Hours and Minutes	0 min per day; Between 1 and 15 min per day; Between 15 and 30 min per day; Between 30 and 60 min per day; Between 1 and 1½ hrs per day; Between 1½ and 2 hrs per day; More than 2 hrs per day
**4**	Thinking about the past week, on a typical day (24 hrs) how much time did your infant spend playing games or using apps on electronic devices such as a computer or laptop, videogame console, iPad, tablet, smartphone, or any electronic gaming device? (e.g. 2 hours 15 minutes)	Hours and Minutes	0 min per day; Between 1 and 15 min per day; Between 15 and 30 min per day; Between 30 and 60 min per day; Between 1 and 1½ hrs per day; Between 1½ and 2 hrs per day; More than 2 hrs per day
**5**	Thinking about your infant's sleep during the past week, how much time did your infant spend in sleep during the NIGHT (between 7 in the evening and 7 in the morning)? (e.g., 2 hours 15 minutes)	Hours and Minutes	Less than 6 hrs per night; Between 6 and 8 hrs per night; Between 8 and 10 hrs per night; Between 10 and 12 hrs per night; Between 12 and 14 hrs per night; More than 14 hrs per night
**6**	Thinking about your infant's sleep during the past week, how much time did your infant spend in sleep during the DAY (between 7 in the morning and 7 in the evening)? (e.g., 2 hours 15 minutes)	Hours and Minutes	Less than 1 hr per day; Between 1 and 2 hrs per day; Between 2 and 3 hrs per day; Between 3 and 4 hrs per day; More than 4 hrs per day

	MBQ-C		
**1**	Thinking about the past week, on a typical weekday (24 hrs) how much time did your child spend playing outdoors?	Hours and Minutes	0 min per day; Between 1 and 30 min per day; Between 30 and 60 min per day; Between 1 and 2 hrs per day; Between 2 and 3 hrs per day; Between 3 and 4 hrs per day; More than 4 hrs per day
**2**	Thinking about the past week, on a typical weekend day (24 hrs) how much time did your child spend playing outdoors?	Hours and Minutes	0 min per day; Between 1 and 30 min per day; Between 30 and 60 min per day; Between 1 and 2 hrs per day; Between 2 and 3 hrs per day; Between 3 and 4 hrs per day; More than 4 hrs per day
**3**	Thinking about the past week, on a typical weekday (24 hrs) how much time did your child spend watching television programs, videos/internet clips or movies on a television, computer or portable/mobile device such as tablet or smartphone?	Hours and Minutes	0 min per day; Between 1 and 30 min per day; Between 30 and 60 min per day; Between 1 and 1½ hrs per day; Between 1½ and 2 hrs per day; More than 2 hrs per day

**4**	Thinking about the past week, on a typical weekend day (24 hrs) how much time did your child spend watching television programs, videos/internet clips or movies on a television, computer or portable/mobile device such as tablet or smartphone?	Hours and Minutes	0 min per day; Between 1 and 30 min per day; Between 30 and 60 min per day; Between 1 and 1½ hrs per day; Between 1½ and 2 hrs per day; More than 2 hrs per day
**5**	Thinking about the past week, on a typical weekday (24 hrs), how much time did your child spend playing games or using apps on electronic devices such as a computer or laptop, videogame console, iPad, tablet, smartphone, or any electronic gaming device?	Hours and Minutes	0 min per day; Between 1 and 30 min per day; Between 30 and 60 min per day; Between 1 and 1½ hrs per day; Between 1½ and 2 hrs per day; More than 2 hrs per day
**6**	Thinking about the past week, on a typical weekend day (24 hrs), how much time did your child spend playing games or using apps on electronic devices such as a computer or laptop, videogame console, iPad, tablet, smartphone, or any electronic gaming device?	Hours and Minutes	0 min per day; Between 1 and 30 min per day; Between 30 and 60 min per day; Between 1 and 1½ hrs per day; Between 1½ and 2 hrs per day; More than 2 hrs per day
**7**	Thinking about the past week, how much time did your child spend in sleep during the NIGHT? (e.g., 2 hours 15 minutes)	Hours and Minutes	Less than 6 hrs per night; Between 6 and 8 hrs per night; Between 8 and 10 hrs per night; Between 10 and 12 hrs per night; Between 12 and 14 hrs per night; More than 14 hrs per night

**8**	Thinking about the past week, how much time did your child spend in sleep during the DAY? (e.g., 2 hours 15 minutes)	Hours and Minutes	0 hrs per day; Less than 1 hr per day; Between 1 and 2 hrs per day; Between 2 and 3 hrs per day; Between 3 and 4 hrs per day; More than 4 hrs per day
**9**	In a typical week, how often does your child have a regular bedtime routine (e.g., bath, story)	Hours and Minutes	Never; 1 - 2 nights per week; 3 - 4 nights per week; 5 - 6 nights per week; Every night

Table S2. Interview outline

"Hi <PARTICIPANT NAME>, Welcome to the Movement Behaviour Questionnaire Project interview today, my name is Denise, thank you for taking the time to participate today."

1. Introduction

"As was explained to you in the online survey, we would like to video record the interview so that I don't have to write down everything you say. Is this still ok?"

***** BEGIN RECORDING HERE *****

- Explain purpose of study:

"The purpose of this study is to better understand how parents interpret survey questions about their child's movement behaviours.

During the interview I will show you some questions about your child's physical activity, screen time and sleep, and ask you to 'think-aloud' while you are reading through the questions.

I will then follow-up with some other questions about your experience of answering the questions. Finally, I will show you some different types of questions and ask you to explain which question format you prefer and why."

  - **ONLY if participant has two eligible children**: *"I can see you have two children…*

…Let's FIRST think about [infant name]" [need to be clear on the child the parent needs to focus on first to provide answers to the directed questions]

OR

…think about JUST ONE of them when you are reading through the questions"

2. Think-aloud - example

"Before we start, I'll ask you a 'warm-up' question to introduce you to the think-aloud process.

You may find it challenging to do this initially, but it will become easier once you have done it a few times as we progress the interview.

Try to visualise the place where you live and think about how many rooms you have. As you count the number of rooms, tell me what you are seeing and thinking about to work out your answer."

3. MBQ – main portion of interview

We will go through the questions together. I want you to tell me what you are thinking to come to your answer. There are no right or wrong answers. We're not focusing on how much physical activity, screen time or sleep your child is doing, but we would like you to provide an answer so that we can better understand how parents think when they're completing surveys about their child's movement behaviours.

Ok let's get started. I will now bring up the survey for you to see."

- ***INTERVIEWER: share screen with first question (skipping intro)***

"The first questions ask about your child's physical activity. Now I would like you to read the first question out loud and tell me what you are thinking as you answer the question."

Prompts for all participants

AT EVERY QUESTION DURING THE INTERVIEW

- *'Is the wording of the question clear?' If NO, why not?*
- *'Is the question difficult to answer?' Why/why not?*

If the question IS NOT clear or not easy to answer:

- *'How would you rephrase the question?'*
- *'What changes would you make to this question to make it clearer?'*

AT LEAST ONCE DURING THE INTERVIEW, PREFERABLY AT THE FIRST INSTANCE

- Direct parent to the **first part of the question** 'Thinking about the past week, on a typical day (24 hours) …' and ask the following:
  - *'Does this part of the question make sense to you?' If NO, why not?*

- **For similar questions only varying by weekday or weekend day,** make sure parent is clear on distinction as they read through the questions by asking:
  - *'Is it clear that the question is asking you about PA on weekdays? Is it clear that the question is asking about PA on weekend days?'*

AT THE END OF THE CLOSED ANSWER OPTION SECTION (screen time, sleep)

Prompts relating to the closed answer option questions:

"For these last few questions in this section on [PA, screen time, sleep] …

- *'Do the answer options make sense to you? If NO, why not AND 'what would you change?'*
- *'Was the response you wanted to provide included in the answer options?'* IF NO, *'what response is needed?'*
- *'Do you think the ranges of the answer response options ok?'* IF NO, *'why not?'* AND *'What ranges would you like to see?'*

Comparison of open/closed formats

- *'Of these questions, is there one format which you prefer or find easier to answer?'* AND *'why?'*

AT THE END OF THE PA and Screen time SECTION, direct parent to the **inclusion of the (24 hrs) in the question**, and ask the following:

- *'Did the inclusion of the 24 hours in this question make you think about any activities OR screen time after dark?'* If YES, which ones?

AT THE END OF THE SCREEN TIME SECTION

- *'Are there are any electronic devices that your child watches or plays with that we have NOT mentioned in the screen time questions?'* If YES, *'Please list which ones.'*

AFTER EACH SET OF SCREEN TIME QUESTIONS (watching/playing, weekday AND weekend day) IN THE SCREEN TIME SECTION

- *'Would your answer change if we asked you to think about times your infant/child watches a screen at home,* ***as well as*** *when you're out and about, such as in the car, at the shops, or at restaurants?'*

- INTERVIEWER NOTE. Prompt participant to think of more usual times if they mention that now (COVID) they are not out and about often.

DURING THE CHILD SURVEY:

AFTER THE FIRST OPEN-ENDED QUESTION (including both weekday and weekend day) IN THE PHYSICAL ACTIVITY SECTION

- *'If we asked you the question this way, would your answer change?' IF YES, 'what is your answer?' AND 'How did you come to that answer?'*

- INTERVIEWER NOTE. Prompt participant to read out loud the next question (i.e. alternate open-ended questions)

AFTER THE SECOND OPEN-ENDED QUESTION (including both weekday and weekend day) IN THE PHYSICAL ACTIVITY SECTION

- *'Is the wording of the question clear?' If NO, why not?*

If the question IS NOT clear:

- *'How would you rephrase the question?'*
- *'What changes would you make to this question to make it clearer?'*

AT THE CHILD SCREEN TIME SECTION

Following the occurrence of QUESTION B) at the end of the 'watching' set and at the end of the 'playing' set ('of this time, how much how much time did they watch/play with/use an electronic device **while standing**?', ask the following:

- *'Compared to Question B) on the screen, would it be easier if we asked the question this way:* 'Of this time, how much time did they watch/play with/use an electronic device while they were **sitting or lying down**?'

AT THE END OF THE CHILD sleep section – comparison of question formats (open vs. closed vs. clock)

- *'Of these questions, is there one format which you prefer or find easier to answer?'* AND *'why?'*

DURING THE INFANT SURVEY:

AFTER THE INFANT TUMMY TIME QUESTION IN THE PHYSICAL ACTIVITY SECTION, if parent says they no longer are doing 'tummy time'

- *'At what age did you stop doing 'tummy time' with your infant?*

AFTER THE INFANT ACTIVE PLAY QUESTION IN IN THE PHYSICAL ACTIVITY SECTION,

- *'Other than 'tummy time', and the activities described in this question, are there any other activities your infant does that you consider to be active play?'*

AT THE END OF THE INFANT SURVEY

- *'Do you have a preference on whether we refer to your child as 'baby' or 'infant'?'*

- **Repeat think-aloud, paraphrasing and probing question process for screen time**
- **Repeat think-aloud, paraphrasing and probing question process for sleep**

  - **ONLY if participant has two eligible children**: *'Let's now think about [child name]'* [need to be clear on the child the parent needs to focus on first to provide answers to the directed questions]

'Ok let's get started. I will now bring up the survey for you to see.'

  - ***INTERVIEWER: share screen & start at first question of next survey***

- **Repeat think-aloud, paraphrasing and probing questions FOR THE other eligible child**

4. Interview close

- Ask if participant if they would like to receive a $20 Thank You gift card. This will be e-mailed.

"Thank you for taking the time to participate in the MBQ Study"
